# Astrocyte-Microglia Crosstalk: A Novel Target for the Treatment of Migraine

**DOI:** 10.14336/AD.2023.0623

**Published:** 2024-05-07

**Authors:** Mingsheng Sun, Jing Rong, Mengdi Zhou, Yi Liu, Shiqi Sun, Lu Liu, Dingjun Cai, Fanrong Liang, Ling Zhao

**Affiliations:** College of Acupuncture and Tuina, Chengdu University of Traditional Chinese Medicine, Chengdu, China

**Keywords:** migraine, astrocyte, microglia, crosstalk, inflammation

## Abstract

Migraine is a pervasive neurologic disease closely related to neurogenic inflammation. The astrocytes and microglia in the central nervous system are vital in inducing neurogenic inflammation in migraine. Recently, it has been found that there may be a crosstalk phenomenon between microglia and astrocytes, which plays a crucial part in the pathology and treatment of Alzheimer's disease and other central nervous system diseases closely related to inflammation, thus becoming a novel hotspot in neuroimmune research. However, the role of the crosstalk between microglia and astrocytes in the pathogenesis and treatment of migraine is yet to be discussed. Based on the preliminary literature reports, we have reviewed relevant evidence of the crosstalk between microglia and astrocytes in the pathogenesis of migraine and summarized the crosstalk pathways, thereby hoping to provide novel ideas for future research and treatment.

## 1. Introduction

A migraine is characterized by severe unilateral headache recurrence and other neurological symptoms. It affects 15.1% of the world’s population and is one of the most common neurological diseases encountered in clinical practice. Inflammation of the trigeminovascular system is one of the leading causes of migraines [1] and provides an anatomical and neurophysiological basis for understanding them better [2]. Previous studies have shown that activated microglia and astrocytes significantly contribute to neurogenic inflammation. Release of pro-inflammatory cytokines can increase arachidonic acid product levels, which can provoke migraine and other neurological manifestations, including fatigue and nausea [3, 4].

As research on astrocytes and microglia has become more profound, the crosstalk between them has become a novel focus in neuroimmunity [5]. It has been thoroughly studied in inflammatory diseases such as Alzheimer's disease (AD) [6]. Microglia and astrocytes can be activated into two polarized states: a pro-inflammatory phenotype (microglia: M1 phenotype and astrocytes: A1 phenotype) and an anti-inflammatory phenotype (microglia: M2 phenotype and astrocytes: A2 phenotype). On the one hand, in a pathological state, microglia (M1) can regulate the activation of astrocytes (A1). The activated astrocytes (A1) can recruit more immune cells to migrate to the injured area, forming a mutual information dialogue and thus facilitating cascade amplification of the inflammatory effect in the initial stage, but on the other hand, the activated astrocytes (A2) can also deter the overreaction of microglia (M1) by releasing multiple cytokines [7]. However, the interaction between astrocytes and microglia in migraines remains unclear. Emerging evidence indicates that targeted regulation of the “astrocyte-microglia” crosstalk can potentially improve neurological function [8, 9]. Novel treatments for various central inflammatory diseases may rely on recognizing the molecular mediators involved in astrocyte-microglia crosstalk and the factors conducive to facilitating the recovery of the appropriate glial phenotype and functional homeostasis. Therefore, we introduced the concept of astrocyte-microglia crosstalk into the field of migraine research and reviewed contemporary research trends with the expectation of further promoting pathological research and treatment of migraines.

## 2. Microglia, astrocytes, and migraines

### 2.1. Microglia and migraines

Microglia are vital immune cells in the central nervous system (CNS) [1] that can sense changes in the microenvironment and respond quickly. Microglia are critical for the brain’s innate immunity. They are the dominant regulator of neuroinflammation and are of great significance in developing, maintaining, and repairing the CNS [10, 11]. Microglia activation is crucial for abnormal neuronal signal transmission; however, abnormal microglia activation can result in migraines and other diseases [12].

According to a recent study, microglia can be divided into two phenotypes: M1 and M2 microglia. M1 microglia (biomarker: interleukin 1 beta (IL-1β), IL-6, tumor necrosis factor-alpha (TNF-α), etc.) [13, 14] are a group of cells that mainly secrete pro-inflammatory cytokines and show pro-inflammatory functions. They can produce TNF-α, IL-1β, IL-6, and other inflammatory mediators, such as glutamic acid and nitric oxide (NO), initiate inflammatory reactions, and lead to apoptosis and secondary injury, showing pronounced neurotoxic effects. M2 microglia (biomarker: IL-10, IL-4Rα, YM-1, etc.) [13, 15] can generate a large number of platelet-derived growth factors (PDGF), transforming growth factor beta (TGF-β), IL-10, and other cytokines; hence, they play a vital role in angiogenesis, anti-inflammatory factor secretion, and inflammatory repair.

The effect of microglia on migraines has gradually gained public attention. A study found that increased P2Y14 receptors in the trigeminocervical complex (TCC) microglia are essential in generating mechanical allodynia in migraine rat models [16]. As a purinergic receptor expressed in microglia, the P2X7 receptor (P2X7R) participates in the central sensitization of chronic migraine (CM) and has become a potential target for its treatment [17]. Microglial glucagon-like peptide-1 receptor (GLP-1R) activation in the trigeminal nucleus caudalis (TNC) may regulate microglial activation in the TNC via the PI3K/Akt pathway to suppress the central sensitization of CMs [18]. A study on migraine rat models [19] found that microglial activation was evident in the cervical spinal cord posterior horns, and microglial inhibitor pretreatment could effectively suppress hyperalgesia and c-Fos expression. Moreover, researchers discovered that [20] microglia in the TNC could be activated by repeated dural stimulation with inflammatory soup (IS), causing region-specific increases in blood-brain barrier (BBB) permeability isolated from the TNC in rats.

### 2.2. Astrocytes and migraines

Astrocytes are the most abundant cell type in the CNS and are mainly responsible for regulating brain homeostasis, including maintaining ion gradients and neurotransmitter clearance [21]. They are a vital link in the pathogenesis of various CNS diseases and are the main targets of many CNS disease treatments [22]. When stimulated by CNS diseases or exogenous substances, astrocytes are activated [23], shifting from their normal resting state to an active state and forming reactive astrocytes characterized by hypertrophy, leading to high expression of intermediate filament proteins and functional changes [24]. During activation, astrocytes secrete various cytokines, chemical molecules, growth factors, and neurotrophic factors [25]. Interleukins, growth factors, and chemokines bind to specific receptors on the astrocyte membrane, activate various signaling pathways in cells, and regulate the transcription and expression of corresponding genes. Therefore, the activation of astrocytes is not controlled by a simple "on-off" pathway but is finely regulated by many intracellular and extracellular signals together [25]. There are two phenotypes of activated astrocytes: the A1 astrocyte (inducer: IL-1α, complement component 1, q subcomponent, TNF-α, etc.; biomarker: complement component C3) [26, 27] that destroys synapses and kills neurons and the A2 astrocyte (biomarker: Clcf1, LIF, IL-6, IL-10, etc.) that has a protective effect on neurons [26]. Astrocytic activation is a double-edged sword. It is indispensable for reducing injuries and the spread of inflammation but also inhibits the regeneration of synapses and cells [28].

According to the latest research [29, 30], astrocytic activation significantly affects central sensitization, which is the potential pathogenesis of migraines. In the IS-induced mouse model, A1 astrocytes are generated and sustained, which may lead to migraines by regulating the release of CC-chemokine ligand 7 (CCL7), CC-chemokine ligand 12 (CCL12), and IL-10. Apart from their connection to central sensitization, gene sets containing genes related to astrocytes also tend to elicit migraines. Furthermore, impaired astrocyte function facilitates migraine-like cranial pain in mouse models [31].

**Figure 1.**
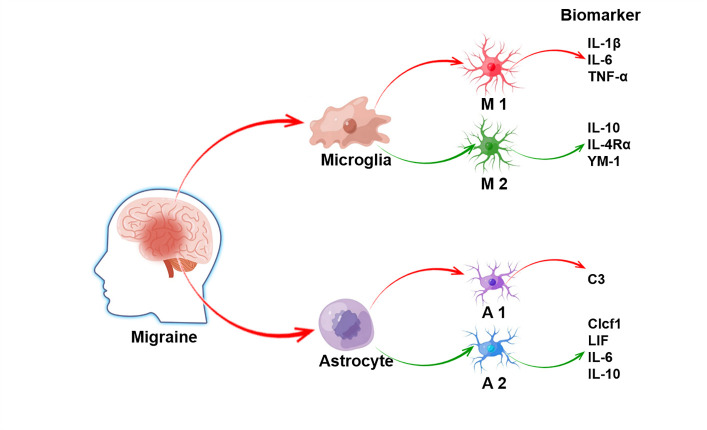
Microglia and astrocyte in migraine.

Therefore, different types of microglia and astrocytes are of great importance in developing and treating migraine. However, their dynamic interactions need further exploration (Fig. 1).

## 3. Pro-inflammatory pathways related to migraine in astrocyte-microglia crosstalk

### 3.1. Inflammatory cytokines crucial to pro-inflammatory astrocyte-microglia crosstalk

Cytokines are vital endogenous substances involved in immune and inflammatory reactions. Neurogenic inflammation caused by changes in cytokine levels is crucial in the pathogenesis of migraine [32]. The activation of microglia and astrocytes exhibits noticeable sequential features. The expression of pro-inflammatory cytokines in microglia reaches its peak 2-4 h after the peripheral injection of lipopolysaccharide (LPS), while cytokine expression in astrocytes is delayed and peaks at 12 h [13]. Astrocytes produce certain pro-inflammatory factors; however, microglia remain a major source of cytokines [27]. Therefore, the inflammatory activation of microglia provides a crucial initiative in the pro-inflammatory process of astrocyte-microglia crosstalk.

#### 3.1.1. Microglia (IL-1)-Astrocyte (IL-1R)

IL-1 is a highly active pro-inflammatory cytokine that lowers pain thresholds and damages tissues. It can be further divided into IL-1α and IL-1β [33]. In the migraine models’ trigeminal ganglion (TG), higher levels of microglial activation and increased production of IL-1β were observed [34]. Previous studies have found that microglia modulate astrocytic activation via the release of IL-1 and that IL-1Rs are mainly expressed in astrocytes [35]. It has been confirmed that blocking the IL-1/IL-1R pathway in the TNC brain region can inhibit pain hypersensitivity in migraine mice[36]. Therefore, when IL-1Rs on astrocytes receive an IL-1 signal from microglia, it can initiate a series of intracellular signal cascade reactions, finally leading to hyperalgesia seen in migraines.

#### 3.1.2. Microglia (IL-18)-Astrocyte (IL-18R)

IL-18 is a vital regulator of innate and acquired immune responses and participates in pain processes, including neuropathic pain. A previous study showed that microglia-derived IL-18 signaling in the medullary dorsal horn is essential in experimental migraine [37]. On the one hand, IS dural infusions induced microglial activation and the up-regulation of IL-18 through the p38 MAPK pathway derived from toll-like receptor 4 (TLR4); on the other hand, microglia-derived IL-18 further acted on IL-18R primarily expressed by astrocytes and increased phosphorylation of nuclear factor-kappa B (NF-κB), thus activating astrocytes. However, blocking the IL-18 signaling pathway can weaken nociceptive behavior and suppress NF-κB phosphorylation and astrocyte activation [37]. It can be concluded that this interaction between microglia and astrocytes mediated by IL-18/IL-18R enhanced tactile allodynia via glial cell-specific NF-κB signal transduction cascades.

#### 3.1.3. Microglia (VEGF-B)-Astrocyte (VEGFR-1)

Vascular endothelial growth factor (VEGF) is a primary regulator of vascular development and blood and lymphatic function and can increase vascular permeability. The VEGF-VEGFR system is a critical link in regulating inflammation [38]. VEGF may play a significant role in migraine pathogenesis and/or chronification in a novel case-control study of patients [39]. Further research found that the effects microglia have on astrocytes can also be targeted: microglia-derived VEGF-B can control astrocytic function concerning CNS pathology, trigger the VEGFR-1 activation in astrocytes, and promote CNS inflammation [10]. Therefore, it may be one of the main signaling pathways of the trigeminovascular system leading to migraine pain symptoms.

#### 3.1.4. Astrocyte (IL-15)-Microglia (IL-15R)

IL-15 is a pro-inflammatory cytokine that coordinates homeostasis and intensity of immune responses. Previous studies have shown that IL-15 can promote immune system activation and nociceptor sensitization in headache diseases such as migraine [40]. The IL-15 glial fibrillary acidic protein promoter (GFAP-IL-15) controls the expression of IL-15. In GFAP-IL-15 mice, astrocyte-targeted IL-15 expression results in neurological deficits. GFAP-IL-15 mice show an accumulation of microglia close to astrocytes in the diseased brain [41]. A human study also found that human microglia mainly express the mRNA transcript of IL-15R [42]. Therefore, IL-15 can be determined as a primary mediator of the pro-inflammatory crosstalk between astrocytes and microglia in migraines.

#### 3.1.5. Astrocyte (IL-33)-Microglia (ST2)

Interleukin-33 (IL-33) is a member of the multifunctional IL-1 family of cytokines [43]. Previous studies have shown that the interaction between IL-33 and microglia mediates pain sensitivity related to migraines [44]. Astrocytes are the primary source of IL-33 in the brain, and microglia mainly express Interleukin 1 Receptor-Like-1 (ST2) [45, 46]. Research has demonstrated that astrocyte-derived IL-33 promotes microglial synapse engulfment, the development of neural circuits, limits the number of excitatory synapses during development by facilitating the engulfment of synaptic proteins [45], and concurrently promotes the increased production of pro-inflammatory cytokines, chemokines, and oxidative stress molecules [43-45]. Therefore, abnormal IL-33/ST2 signaling pathway activation can aggravate the central sensitization of migraine and other diseases.

### 3.2. Chemokine crucial to pro-inflammatory astrocyte-microglia crosstalk

Chemokines are a kind of small molecular protein (8-10 kDa) with chemotactic activity belonging to the cytokine family, which can mediate the migration of leukocytes to inflammatory sites. They are expressed in large amounts in many regions of the brain and spinal cord [47]. Chemokines perform various functions in the CNS during development. Throughout adulthood, they continue to mediate intercellular communication [48] and are closely associated with CNS inflammatory diseases [49] and pain [50]. In a study investigating chemokine levels in patients with migraines, increased levels of chemokines were found both in the interictal period and during attacks [51], suggesting that chemokines may be related to their pathogenesis.

#### 3.2.1. Astrocyte (CCL2)-Microglia (CCR2)

CC chemokine ligand 2 (CCL2) is from the C-C motif chemokine family. It is of great significance and a key mediator in inflammation. Furthermore, it can also attract or enhance the expression of other inflammatory factors/cells [52]. The astrocyte-derived chemokine CCL2 is crucial in microglial activation and movement during inflammation and subsequent neurodegeneration, and the CCL2/CCR2 axis is involved in astrocyte-microglia crosstalk, which mediates microbial activation and thus contributes to increased neuroinflammation [53]. According to previous clinical research, a high level of MCP-1/CCL2 was observed in the serum of patients with migraine [54]; moreover, CCR2 was also associated with migraine [55]. The CCL2-CCR2 pathway participates in CNS inflammation during migraine to a large extent.

### 3.3. Complement system crucial to pro-inflammatory astrocyte-microglia crosstalk

The complement system is part of the innate immune system and enhances the engulfment of cellular debris, apoptotic bodies, and misfolded proteins [56]. In a healthy brain, the complement system can influence neurodevelopment, neurogenesis, synaptic pruning, clearance of neuronal vesicles, recruitment of phagocytes, and protection from pathogens. A study on the serum proteomic profile of menstrual-related migraines showed upregulation of complement compared with non-headache control females [57].

#### 3.3.1. Microglia (C1q)-Astrocyte (C1qR)

As a novel adipocyte factor, the q subcomponent (C1q) may participate in metabolism and inflammation [58]. Abnormal microglial activation can induce A1 reactive astrocytes in vitro and in vivo. It has also been demonstrated that A1 reactive astrocytes are mainly induced by microglia-derived cytokines (such as C1q) [26]. In a study on migraines [59, 60], it was found that patients with a genetic defect in the complement 1 inhibitor manifested migraine-like episodes, and using a C1 esterase inhibitor (C1-INH) could block the C1q-C1qR pathway to improve migraine disability. Therefore, the C1q-C1qR pathway may mediate crosstalk between microglia and astrocytes to produce migraine pain symptoms.

#### 3.3.2. Astrocyte (C3)-Microglia (C3aR)

C3 is a protein in the complement system conducive to the innate immune response. C3/C3aR signaling is vital to innate immune pathogen defense and plays a considerable role in inflammation and neurodegeneration [61]. Novel proteomics analysis revealed that the expression levels of complement C3 were higher in patients with migraine than in healthy volunteers or during pain than in the pain-free period [62]. Animal studies have found that in a mouse model of CNS inflammatory disease, whose complement pathway has been activated, complement factor C3 secreted by astrocytes interacts with the microglial C3a receptor (C3aR) to regulate microglial engulfment dynamically [8]. Therefore, it can be concluded that the C3/C3aR complement-activated astrocyte-microglia crosstalk is the core of neuroinflammation in a mouse model of CNS inflammatory diseases, such as migraine.

### 3.4. Lipocalin proteins crucial to pro-inflammatory astrocyte-microglia crosstalk

Lipocalin proteins are adipocyte factors mainly expressed in mononuclear phagocytes, neutrophils, and adipocytes and are closely related to obesity, chronic inflammation, and other diseases. Lipocalin proteins released by astrocytes and related microglia-mediated signaling molecules regulate neuroplasticity [63].

#### 3.4.1. Astrocyte (LCN2)-Microglia (LCN2R)

Lipocalin-2 (LCN2) is an inflammatory protein proven to be involved in CNS diseases and their risk factors. Experimental studies have shown that LCN2 affects various neuropathophysiological processes, including aggravating neuroinflammation, cell death, and iron dysregulation [64, 65]. Compared with the control group, the serum LCN2 level in patients with migraine was significantly higher, and a positive correlation was observed between the visual analog scale score, the number of days with pain, and the LCN2 level [66]. LCN2 is another mediator of astrocyte-microglia interactions in disease conditions, amplifying microglial activity during CNS inflammation and pathological conditions [67]. LCN2 is expressed in astrocytes, whereas the LCN2 receptor (LCN2R) is mainly expressed in microglia [63]. Therefore, LCN2R is expressed in microglia, indicating that astrocyte-derived LCN2 may act on microglia, stimulate crosstalk, and regulate migraines and other CNS neuroinflammatory diseases.

## 4. Anti-inflammatory pathways related to migraines in astrocyte-microglia crosstalk

### 4.1. Inflammatory cytokines crucial to anti-inflammatory astrocyte-microglia crosstalk

#### 4.1.1. Astrocyte (IL-3)-Microglia (IL-3Rα)

Astrocyte-derived IL-3 programs microglia to improve the pathology of nervous system inflammation. Moreover, microglia will increase expression of IL-3Ra (IL-3 specific receptor, also known as CD123) so that they may respond to IL-3. Astrocytes can specifically produce IL-3, which can trigger the reprogramming of transcription, morphology, and function, endowing them with an acute immune response program, strengthened motility, and the capability to cluster and clear Aβ and tau aggregates. It has been established that IL-3 is the central mediator of astrocyte-microglia crosstalk and the node for therapeutic intervention of nervous system inflammation [6]. Furthermore, in transgenic mice, IL-3 was indispensable for generating multiple sclerosis (MS)-like disorders in brain macrophages, and neurological dysfunction were observed in mice expressing antisense IL-3 RNA [68]. The cortical spreading depression (CSD) is believed to be a connection between MS and migraines, considering that cortical demyelination closely related to MS may accelerate CSD and initiate migraines [69]. Accordingly, fluctuations in IL-3 levels may be an essential factor in migraine treatment.

#### 4.1.2. Microglia (IL-10)-Astrocyte (IL-10R)

IL-10 is a critical anti-inflammatory cytokine that alleviates inflammatory pain and is produced by T (Treg) [70]. Multiple linear regression models show that migraines are positively correlated with TNF-α and negatively related to IL-10 [71]. Activated M2 microglia produce the anti-inflammatory cytokine IL-10, matching the IL-10 receptor (IL-10R) mainly expressed in A2 astrocytes, enabling them to produce TGF-β to reduce microglia activation [72]. This suggests a novel mechanism of astrocyte-microglial regulation under inflammatory conditions [73]. It has also been confirmed [32] that the interictal level of IL-10 in migraine patients decreases, indicating that IL-10 is closely related to migraine remission. A later study further pointed out that IL-10 signaling pathways in trigeminal ganglions are potential targets for migraine treatment [74]. Therefore, the IL-10/IL-10R pathway may be a promising way to inhibit migraine pain symptoms by regulating crosstalk between microglia and astrocytes.

### 4.2. Neurotransmitters crucial to anti-inflammatory astrocyte-microglia crosstalk

Neurotransmitters are chemicals that transmit information between nerve cells or between nerve cells and effector cells such as myocytes and gland cells. Previous studies have shown that migraines are closely related to various neurotransmitters, including adenosine triphosphate (ATP) and glial cell-derived neurotrophic factor (GDNF) [75-78].

**Figure 2.**
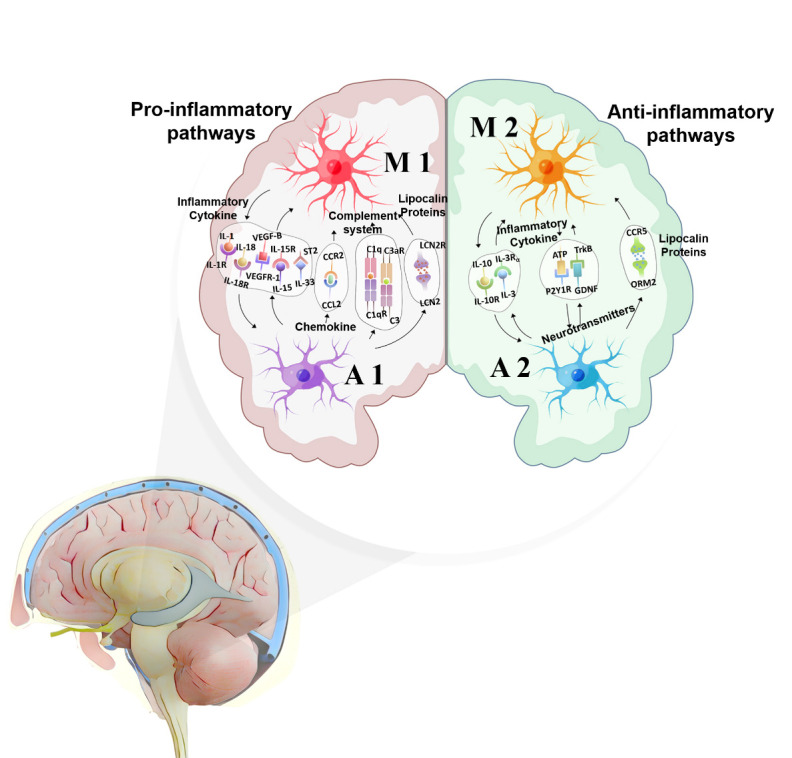
Pro-inflammatory pathways and anti-inflammatory pathways related to migraines in astrocyte-microglia crosstalk of the trigeminovascular system

#### 4.2.1. Astrocyte (GDNF)-Microglia (TrkB)

GDNF and brain-derived neurotrophic factor (BDNF) are vital astrocyte-releasing molecules that regulate microglial activation. A clinical study reported that a decline in GDNF could lead to persistent central sensitization of CM [78]. It has been reported that astrocyte-derived GDNF controls the receptor tropomyosin-related kinase B (TrkB) in microglia, modulates microglial activation, and prevents neurodegeneration by inhibiting neuroinflammation [79-81]. Overexpression of BDNF in the ventrolateral periaqueductal gray (vlPAG) decreases the severity of epileptic and migraine-like events in comorbid rats, indicating an analgesic effect by activating the BDNF-TrkB signaling pathway [82]. Therefore, the GDNF-TrkB signaling pathway may improve migraine TNC and trigeminal ganglion inflammatory conditions.

#### 4.2.2. Microglia (ATP)-Astrocyte (P2Y1R)

ATP and its derivatives initiate and propagate migratory signals through several mechanisms. For example, they participate in vasomotor mechanisms, CSD, and fast transmission or cross-excitation based on satellite glial cells in the trigeminal ganglion [83]. In migraine models, ATP administration triggers BDNF release, increases BDNF synthesis in the TNC, and decreases central sensitization symptoms [82, 84]. Previous studies found that activated microglia can release ATP, activating the reactive phenotype of astrocytes [85] and reducing neuronal damage with the activation of astrocytic P2Y1R [86, 87]. P2Y1R-KO mice display a high number of injured neurons [88]. Therefore, the astrocyte P2Y1R activated by microglial ATP may be neuroprotective and can be used to treat migraines.

### 4.3. Lipocalin proteins crucial to anti-inflammatory astrocyte-microglia crosstalk

#### 4.3.1. Astrocyte (ORM2)-Microglia (CCR5)

Orosomucoids (ORM) belong to the immunocalin subfamily, a group of small molecule-binding proteins with immunomodulatory functions. Under inflammatory stimulation, ORM2 protein is primarily expressed and secreted by astrocytes [89]. In the late stage of inflammation, ORM2 released by astrocytes binds to CC-chemokine receptor 5 (CCR5), and further regulation of microglial activation exerts an anti-inflammatory effect, indicating that ORM2 interacts with microglia as a novel mediator in astrocytes [89]. Network pharmacology studies have found that CCR5 regulation may have a therapeutic effect on migraines [90]. Therefore, the ORM2-CCR5 signaling pathway may also be a potential target for treating migraines (Fig 2).

## 5. Conclusion and Perspectives

Migraine is a common paroxysmal brain disease, and a crucial part of its pathogenesis is central sensitization caused by the abnormal activation of microglia and astrocytes. The interaction between microglia and astrocytes significantly influences the neuroinflammatory state of migraines, which mainly depends on their different phenotypes and functions. Local extracellular and intracellular signals determine cell characteristics and phenotypic transformations. The typical inflammatory activation pathway of astrocyte-microglia crosstalk is that microglia are usually more sensitive to pathogens or injuries and are activated to the M1 phenotype through molecular patterns related to injuries or pathogens, which promotes the secretion of inflammatory factors such as IL-1 and IL-18, subsequently activating related receptors on astrocytes (A1 phenotype) and facilitating neuroinflammation [26, 91]. On the other hand, activated A1 astrocytes can secrete inflammatory factors such as IL-15 and IL-33, as well as chemokines (MCP-1/CCL2, CXCL12), complement (C3) and LCN2, which further activates the microglial M1 phenotype, and the cascade amplifies the inflammatory reaction. However, with the limitations of injury and vascular and neural remodeling, local environmental factors change, resulting in the phenotypic transition of microglia and astrocytes. Activated M2 microglia can produce the anti-inflammatory cytokine IL-10 and neurotransmitter ATP that match with the IL-10R and P2Y1R, which are mainly expressed in astrocytes, and activate A2 astrocytes, thus making astrocytes secrete TGF-β and reducing microglial activation [92]; the activated A2 astrocytes can also secrete IL-3, a neurotransmitter (GDNF) and lipocalin proteins (ORM2), which act on M2 microglia, promote neuronal repair, reduce inflammation and alleviate pain, forming a classic anti-inflammatory activation pathway of astrocyte-microglia crosstalk. This study constructs a comprehensive paradigm for astrocyte-microglia crosstalk in migraine research.

Regarding treatment, many existing treatment methods have been found to play therapeutic roles because of astrocytes, microglia, and the interplay between them. Various human and animal studies have found that inhibiting microglial activation by minocycline can alleviate depressive symptoms, and many psychiatric approaches (ketamine, mirtazapine, fluoxetine, and repetitive high-frequency transcranial magnetic stimulation) on astrocytes have a favorable effect and improve depressive symptoms, indicating the vital role of astrocytes and microglia in depression [93]. Likewise, through interactions with microglia in multiple ways, including direct and indirect interactions with astrocytes, melatonin plays a part in the anti-inflammatory process under different grades of brain inflammation [94]. Aerobic exercise is a potentially conducive strategy for preventing migraines [95]. A mechanism study elucidated that [81, 96, 97] physical exercise could act on astrocyte-microglia crosstalk through IL-1, enhancing neuroplasticity and triggering the neuroplastic effect of exercise.

Additionally, C3, MCP-1, TNF-α, and C1q are also included in the motion-induced inflammatory molecules mediating astrocyte-microglia crosstalk. Acupuncture is also a potential intervention for astrocyte-microglial crosstalk. Previous research shows that [98, 99] acupuncture can inhibit the abnormal activation of microglia and astrocytes, increase the mechanical pain threshold, and alleviate the occurrence of mechanical pain sensitization, suggesting that the analgesic effect of acupuncture may be achieved by inhibiting the activation of central glial cells and the inflammatory reaction through the key link of astrocyte-microglia crosstalk. However, the action pathways of the above treatment methods in astrocyte-microglia crosstalk remain unclear. Therefore, astrocyte-microglia crosstalk may become a new avenue that promotes progress in migraine pathology and treatment.

Recently, through high-throughput flow cytometry screening and single-cell RNA sequencing, researchers have recently identified a new subset of astrocytes in mice that mainly expresses the lysosomal protein LAMP1 and the death receptor ligand TRAIL. This astrocyte subset can induce the apoptosis of effector T cells through TRAIL-DR5 signaling, thus limiting inflammation in the CNS [100]. This provides a new idea for discovering new cell subsets in the astrocyte-microglia crosstalk. The single-cell and spatial transcriptomes are the latest methods of biological information analysis, and single-cell omics provide a great convenience for understanding biological heterogeneity and solving biological problems at a higher resolution. Spatial transcriptomes can preserve the spatial positions of tissues and analyze the transcriptomic data of tissue slices. In a study on intercellular communication [101], the interaction of different types of cells was inferred from transcriptomic data and known ligand-receptor complexes [102-104]. It can locate and distinguish the expression of functional genes in specific spatial positions, identify the spatial heterogeneity of particular and different cell subsets in the brain, and obtain information such as the morphology, activity, or connectivity of these subsets. Through these two novel technologies, the seeded region-cell-cell interaction analysis can be carried out in-depth to construct an atlas of the dynamic cellular environment that drives the development and treatment of diseases [105]. In the latest discussion on the nomenclature of microglia by a group of multidisciplinary experts, it is also believed that the new nomenclature of microglia, including the characteristics of single-cell RNA sequencing, will help in better identification [106]. Therefore, new technologies such as single-cell omics and the discovery of new classifications of microglia and astrocyte subtypes can provide a more specific solution for studying astrocyte-microglia crosstalk in migraine.

Overall, we discussed several pathways of the astrocyte-microglia crosstalk closely related to inflammation in the trigeminovascular system in the pathogenesis and treatment of migraine. This review will provide valuable ideas for the discovery of novel potential targets for the treatment of migraine. At the same time, the continuous construction of new classification systems of astrocyte and microglia subtypes will provide the possibility for a more accurate understanding of astrocyte-microglia crosstalk.
